# GABNet: global attention block for retinal OCT disease classification

**DOI:** 10.3389/fnins.2023.1143422

**Published:** 2023-06-02

**Authors:** Xuan Huang, Zhuang Ai, Hui Wang, Chongyang She, Jing Feng, Qihao Wei, Baohai Hao, Yong Tao, Yaping Lu, Fanxin Zeng

**Affiliations:** ^1^Department of Ophthalmology, Beijing Chaoyang Hospital, Capital Medical University, Beijing, China; ^2^Medical Research Center, Beijing Chaoyang Hospital, Capital Medical University, Beijing, China; ^3^Sinopharm Genomics Technology Co., Ltd., Changzhou, Jiangsu, China; ^4^AI-Farm (Nanjing) Big Data Services Co., Ltd., Nanjing, China; ^5^Department of Clinical Research Center, Dazhou Central Hospital, Dazhou, Sichuan, China; ^6^Department of Big Data and Biomedical AI, College of Future Technology, Peking University, Beijing, China

**Keywords:** retinal OCT, retinal disease classification, attention mechanism, model visualization, GABNet

## Abstract

**Introduction:**

The retina represents a critical ocular structure. Of the various ophthalmic afflictions, retinal pathologies have garnered considerable scientific interest, owing to their elevated prevalence and propensity to induce blindness. Among clinical evaluation techniques employed in ophthalmology, optical coherence tomography (OCT) is the most commonly utilized, as it permits non-invasive, rapid acquisition of high-resolution, cross-sectional images of the retina. Timely detection and intervention can significantly abate the risk of blindness and effectively mitigate the national incidence rate of visual impairments.

**Methods:**

This study introduces a novel, efficient global attention block (GAB) for feed forward convolutional neural networks (CNNs). The GAB generates an attention map along three dimensions (height, width, and channel) for any intermediate feature map, which it then uses to compute adaptive feature weights by multiplying it with the input feature map. This GAB is a versatile module that can seamlessly integrate with any CNN, significantly improving its classification performance. Based on the GAB, we propose a lightweight classification network model, GABNet, which we develop on a UCSD general retinal OCT dataset comprising 108,312 OCT images from 4686 patients, including choroidal neovascularization (CNV), diabetic macular edema (DME), drusen, and normal cases.

**Results:**

Notably, our approach improves the classification accuracy by 3.7% over the EfficientNetV2B3 network model. We further employ gradient-weighted class activation mapping (Grad-CAM) to highlight regions of interest on retinal OCT images for each class, enabling doctors to easily interpret model predictions and improve their efficiency in evaluating relevant models.

**Discussion:**

With the increasing use and application of OCT technology in the clinical diagnosis of retinal images, our approach offers an additional diagnostic tool to enhance the diagnostic efficiency of clinical OCT retinal images.

## 1. Introduction

The retina, the visual component responsible for sensing light stimuli, is a soft and transparent membrane located adjacent to the inner surface of the choroid, play an essential role in human vision (Zrenner, 2002). In recent years, there has been a significant increase in the number of patients with retinal disease, which can cause irreversible damage to vision and, in severe cases, result in blindness. Age-related macular degeneration (AMD) and diabetic macular edema (DME) are most common retina diseases (Das, 2016). AMD is divided into two types depending on its clinical presentation and pathological changes: dry AMD and wet AMD. In the early stages of dry AMD, yellowish-white, rounded vitreous warts (drusen) of varying sizes can be seen in the posterior pole of the eye. Wet AMD induces the outward growth of new blood vessels in the choroidal capillaries (known as choroidal neovascularization, CNV). All these pathological features could be important diagnostic indicators of disease progression.

Optical coherence tomography (OCT) is currently the most advanced technique for the detection of retinal diseases, with the advantages of being a noncontact, noninvasive and fast imaging method (Chen and Lee, 2007). The use of OCT technology for fundus images has become widespread, leading to an improvement in the clinical diagnosis of retinal diseases. However, the recognition capability of retinal OCT images varies greatly between different regions, particularly in developing countries where there is a significant shortage of expert physicians compared to the number of patients. Therefore, there is a need to develop an automated machine that can perform recognition on retinal OCT images, reducing the workload of specialist physicians.

Due to the outstanding performance of deep learning in image recognition, more and more researchers have begun to explore its applications in the medical field. However, there are still some challenges in applying deep learning to medical image classification, such as the problem of obtaining enough standardized medical images. In view of this, many researchers have proposed different solutions, among which transfer learning is one of the most commonly used methods. Transfer learning is a machine learning method that is used to correlate two different tasks. Specifically, the parameters of a model trained in one task are transferred to the same model in another task. Since case sourcing in the medical field is extremely difficult and medical image recognition based on deep learning requires a large number of cases to achieve a good classification effect. Transferred model utilizing a model trained on a public dataset can effectively alleviate the difficulty of collecting a dataset in the medical field. A large number of researchers have also used transfer learning in the medical field to obtain good classification results (Narayan Das et al., 2022). Chougrad et al. (2018) developed a computer-aided diagnosis system based on deep convolutional neural networks (DCNNs) to help radiologists classify mammographic occupancy lesions. Kassem et al. (2020) proposed a highly accurate model for the classification of skin lesions, utilizing transfer learning and GoogleNet for pre-training. The initial values for the model parameters are used as before, and they are subsequently adjusted during the training process in order to achieve the best capability to classify various types of skin lesions.

Inspired by the human vision system, which can efficiently view focal regions in complex scenes, a variety of plug-and-play attention mechanisms have been investigated in computer vision (Woo et al., 2018; Hu et al., 2020; Hou et al., 2021) and are widely used in multiple computer vision tasks (Aditya et al., 2021; Wang et al., 2022). Liang et al. (2020) proposed a semi-supervised classification approach based on a CNN model and introduced an attention mechanism to balance the sample weights. Additionally, the focal loss was used to alleviate the poor training effect caused by uneven samples. Farag et al. (2022) proposed a new automatic deep learning-based method for the detection of diabetic retinopathy (DR) severity, first using DenseNet169 as a feature extractor and then introducing a convolutional block attention module (CBAM) on top of it to enhance its discriminative power. Finally, the approach was tested on an external real-world dataset, resulting a good classification capability. Deng et al. (2020) produced a benchmark dataset of breast density images divided into four classes: A (fatty), B (fibrous gland), C (uneven dense), and D (dense). The method begins with data enhancement and normalization of breast images, followed by the implementation of a squeeze-and-excitation (SE) attention module. This module helps to recalibrate the image features and improve the classification of breast density.

Many scholars have also conducted researches on the issue of retinal OCT images. Liu et al. (2011) used the global image descriptor and local binary pattern histogram formed by a multiscale spatial pyramid as a feature vector, which could encode the texture and shape information of a retinal OCT image and its edge image, respectively. Sotoudeh-Paima et al. (2022) proposed a multiscale automated method for classifying AMD-related retinal lesions. Their multiscale CNN architecture was designed by feature fusion based on a feature pyramid network architecture, enabling end-to-end training and reducing the computational complexity of the model compared to that of using multiple CNNs in parallel. Fang et al. (2019) proposed a novel iterative fusion CNN method for retinal OCT image classification. In order to exploit the information between different convolutional layers, the proposed method introduces an iterative layer fusion strategy. Specifically, features from the current convolutional layer are iteratively combined with those from all previous layers in the CNN. Experimental results show that iteratively combining feature information from all layers can achieve better classification results.

Therefore, the challenges in constructing a lightweight network model for retinal OCT grading tasks are as follows.

(1) How to create a plug-and-play attention module.
(2) How to efficiently apply an attention module into a classification network model.

## 2. Related work

### 2.1. Previous studies on retinal OCT image analysis

In recent years, a large number of researchers have been working on retinal OCT analysis, which can be broadly divided into two directions: machine learning methods based on semi-automatic feature extraction and deep learning methods based on fully automatic feature extraction.

Machine learning methods based on semiautomatic feature extraction can be divided into two types: feature extraction and classifier design techniques. The main feature extraction methods are local binary patterns (LBPs), histograms of oriented gradients (HOGs) and scale-invariant feature transform (SIFT) (Lee et al., 2015). The main classifiers are random forests, support vector machines, multilayer perceptron, naive bayes, etc. Srinivasan et al. (2014) designed a method to classify OCT retinal lesion images based on the HOG extracted feature to classify healthy retina, AMD, and DME, achieving high classification accuracy. While machine learning approaches have exhibited promising outcomes in classifying retinal OCT images in recent years, they are accompanied by certain limitations. First, machine learning methods based on semi-automatic feature extraction require manual operations, making them time-consuming and unable to guarantee the quality of the results. In addition, the inconsistency in retinal OCT feature extraction among experts in different regions yields incongruity, thus resulting in inaccurate diagnosis, thereby questioning the veracity of classification outcomes generated by the classifier.

A deep learning approach based on fully automated feature extraction for end-to-end retinal OCT image grading was developed (Das et al., 2021). Kayadibi and Güraksin (2023) used a stacked ensemble learning approach based on CNNs to detect DME, vitreous warts and CNV disease in OCT images. First, features were extracted from OCT images using a fine-tuned AlexNet and then applied to classify using homogeneous and heterogeneous stacked ensemble learning methods.

Semi-automatic feature extraction-based retinal OCT classification methods exhibit certain limitations, including intricate feature engineering and inadequate classification accuracy. While deep learning-based automatic feature extraction has several advantages and can achieve end-to-end prediction effects in retinal OCT disease classification, the current retinal OCT-based deep learning network models suffer from several limitations, including a large number of network parameters and slow model training.

### 2.2. Attention mechanisms

**Figure 1:**
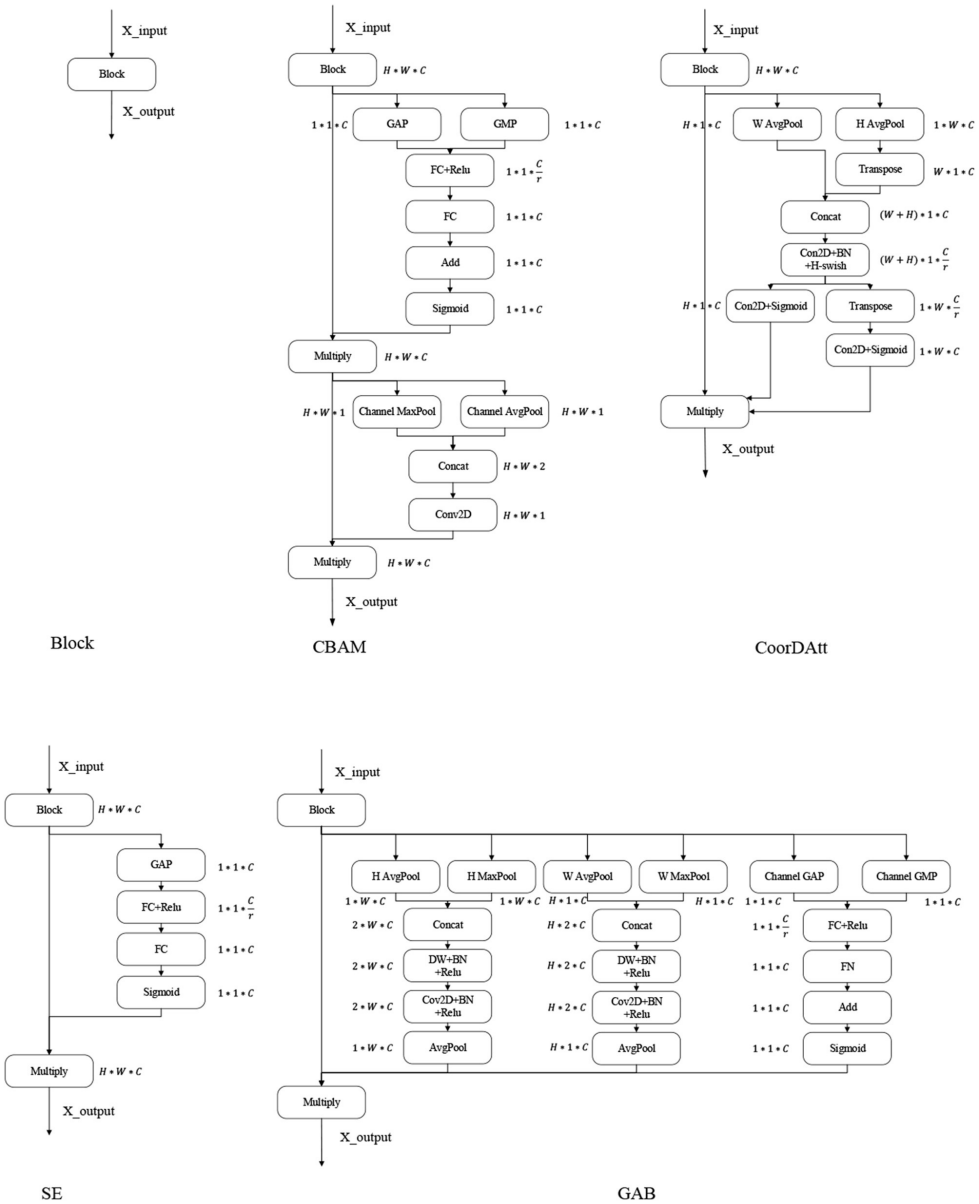
Attention mechanism. GAP, global average pooling layer; GMP, global max pooling layer; Channel AvgPool and Channel MaxPool represent the average and maximum pooling operations conducted on the channels, respectively; W AvgPool and W MaxPool represent the maximum and average pooling operations conducted on the width, respectively; H AvgPool and H MaxPool represent the maximum and average pooling operations conducted on the height, respectively; FC, fully connected layer; ReLU, sigmoid and H-swish are the activation functions; BN, batch normalization; Conv2D, convolutional layer; Add, element-by-element addition; Multiply, vector multiplication; Transpose, transposition of high-dimensional vectors; Concat, stitching according to a certain dimension.

SE (Hu et al., 2020) is a new generic network module architecture unveiled by the autonomous driving company Momenta in 2017 (SE is shown in Figure 1). It models the correlations between feature channels and enhances important features to achieve improved accuracy. The addition of this structure also resulted in an error rate of 2.251% in the top-5 ILSVR competition. SE has been used by a large number of researchers in various industries since its introduction (XinSheng and Yu, 2022). Zhang et al. (2022) proposed a MobileNetV2-SENet-based approach to identify fish foraging behavior. Firstly, the fish images were pre-processed with some operations in order to enhance sample diversity. Then, MobileNetV2 was used to extract fish image features, and an SENet-based feature weighting network was built. Weights were assigned to features with different values. A linear classifier was used to identify the feeding behaviors of the fish. Finally, a method was provided to determine the amount of feeding based on the identification results to reduce feed consumption. Li et al. (2020) fused a DenseNet with SENet modules as the basic classification framework, and conducted extensive experiments on the proposed framework with the public BreakHis dataset, demonstrating the effectiveness of the proposed framework. Yan and Hua (2020) proposed a deep residual SENet (R-SENet) for leaf recognition. The R-SENet employs an SE strategy to learn and obtain the importance level of each channel in each convolutional layer of the residual block to accomplish the recognition task. Subsequently, the weight of each channel is readjusted by the importance level to promote relevant channels and suppress unimportant ones.

CBAM (Woo et al., 2018) is a lightweight convolutional attention module published in the ECCV conference by Woo et al. in 2018 that combines both channel attention module (CAM) and the spatial attention module (SAM) (Figure 1 shows the CBAM). CAM and SAM pay attention to channels and space, respectively. This procedure not only saves parameters and computational power but also ensures that the CAM and SAM can be integrated into existing network architectures as plug-and-play modules. Since its introduction, the CBAM has been used by a large number of researchers (Chen et al., 2020; Li et al., 2021). Luo and Wang (2021) proposed a neural network algorithm incorporating the CBAM in the ResNet architecture by adding the residual blocks of the attention module in the second to fifth layers of the ResNet architecture. The results were finally output by adaptive average pooling and fully connected layers.

Coordinate attention for efficient mobile network design (CoorDatt) is a new attention mechanism designed for lightweight networks that was presented by Hou et al. (2021) at CVPR in 2021 (Figure 1 presents CoorDatt), this mechanism embeds location information into channel attention. Unlike channel attention, which converts the given feature tensor into individual feature vectors via 2-dimensional global pooling, coordinate attention decomposes channel attention into two 1-dimensional feature encoding processes that aggregate features along two spatial directions so that remote dependencies can be captured along one spatial direction while retaining accurate location information. Since the introduction of the CoorDatt module, it has been used by a large number of researchers (Shi et al., 2022; Xiang et al., 2022). Dai et al. (2022) proposed a method for tunnel crack identification based on an improved You Only Look Once version 5 (YOLOv5) architecture. First, tunnel cracks are labeled according to a novel labeling method that uses one labeling box for regular cracks and multiple boxes for irregular cracks. Second, various data augmentations are used to improve the generalization capability of the model. Third, training YOLOv5 in combination with the CoorDatt module can achieve higher tunnel crack recognition rates. Zha et al. (2021) proposed the YOLOv4_MF model. The YOLOv4_MF model utilizes MobileNetV2 as the feature extraction block and replaces the traditional convolution operation with depthwise-separable convolution (DSC) to reduce the number of model parameters. In addition, a coordinate attention mechanism is embedded in MobileNetV2 to enhance feature information. A symmetric structure consisting of a three-layer spatial pyramid pool is proposed, and an improved feature fusion structure is designed to fuse the target information. For the loss function, the focal loss is used instead of the cross-entropy loss to enhance the network's ability to learn small targets.

SE only considers the internal channel information in the feature map and ignores the importance of location information, while the spatial structures of targets in vision are quite important; the CBAM tries to introduce location information by perform global pooling on the channels, but this approach can only capture local information and not long range-dependent information; the CoorDatt approach encodes spatial information through maximum pooling in the horizontal and vertical directions, then transforms it, and finally fuses the spatial information by weighting it over the channels. This approach is not able to resolve the information differences between features in terms of heights, widths, and channels, without the ability to aggregate the information across dimensions. The GAB encodes the height, width and dimension of each data point on the channel of the input feature map. To reduce the number of attention mechanism parameters, we use DSC instead of normal convolution operations.

As shown in Figure 1, the maximum pooling and average pooling operations are first performed on the height and width, and then the DSC operation is performed on them to extract the spatial information of the given feature map from different angles. The obtained feature map is concerned with the detailed information in the image. In this paper, the channel operations are similar to those of the CBAM, where the importance of each channel is learned through attention mechanisms. Additionally, global average pooling is applied to obtain global feature maps, which focus the significant information of interest in the input image.

This paper presents three innovative research content to tackle the limitations of semi-automatic feature extraction, enhance deep learning-based feature extraction methods, and fully exploit the potential of attention mechanisms. The objective of these research content is to improve the accuracy and efficiency of retinal OCT disease classification.

(1) A new attention mechanism module, a global attention block (GAB), is constructed.
(2) A new lightweight classification network model based on the GAB is constructed.
(3) This network is validated on both internal and external retinal OCT datasets and a fundus photograph dataset with DR.

## 3. GAB based on retinal OCT disease classification

### 3.1. System architecture

**Figure 2:**
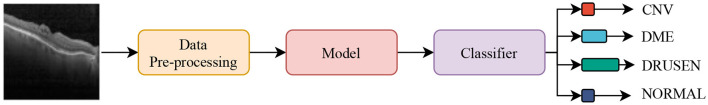
System architecture.

In this study, a classification model consisting of a GAB for retinal OCT disease classification is proposed. The classification model architecture is divided into two modules: a retinal OCT image preprocessing module, and a model training and prediction module. The system architecture is shown in Figure 2. The image preprocessing module mainly includes image data normalization and scaling operations, which are used to unify retinal OCT images of different sizes and facilitate the training of the network. The model training and prediction module constructs the algorithm model and compares predictions in terms of various evaluation metrics. This module is mainly used to train and predict models from the unified retinal OCT images and thus to test the strengths and weaknesses of various algorithmic models.

### 3.2. Image preprocessing module

In the dataset preprocessing module, the input images are mainly deflated to widths of 299 and heights of 299 (by bilinear interpolation) and subsequently normalized (formula 1) to facilitate the calculation of the network.


(1) $$\begin{array}{l} X_{norm}=\frac{X-X_{min}}{X_{max}-X_{min}} \end{array}$$

*X*_*min*_ and *X*_*max*_ represent the minimum and maximum image pixel values, respectively, and *X*_*norm*_ denotes the normalized image pixel values. The normalized image pixel values are restricted to be between 0 and 1, which accelerates the convergence of the neural network and ensures faster convergence when the program is running.

### 3.3. Model training and prediction module

#### 3.3.1. GAB

Given a feature map *T*^H*W*C^, H, W, and C represent the dimensional information of the feature map T (height, width and number of channels, respectively), and m, n, and k represent the size of the feature map (height, width and number of channels, respectively), so the average pooling results in terms of the height and width are obtained via formula 2 and formula 3, respectively.


(2) $$\begin{array}{l} AvgPool\text{\_}H=\frac{1}{m}\overset{m}{\underset{i=1}{\sum }}T_{i}\left(W,C\right) \end{array}$$

(3) $$\begin{array}{l} AvgPool\text{\_}W=\frac{1}{n}\overset{n}{\underset{i=1}{\sum }}T_{i}\left(H,C\right) \end{array}$$

The maximum pooling height and width are shown in formula 4 and formula 5, respectively.


(4) $$\begin{array}{l} MaxPool\text{\_}H=max\left(T_{0}\left(W,C\right),T_{1}\left(W,C\right),T_{2}\left(W,C\right),...,T_{m}\left(W,C\right)\right) \end{array}$$

(5) $$\begin{array}{l} MaxPool\text{\_}W=max\left(T_{0}\left(H,C\right),T_{1}\left(H,C\right),T_{2}\left(H,C\right),...,T_{n}\left(H,C\right)\right) \end{array}$$

The global average pooling process conducted on the channels and global maximum pooling are presented in formula 6 and formula 7, respectively.


(6) $$\begin{array}{l} GAP=\frac{1}{m*n}\overset{m}{\underset{i=1}{\sum }}\overset{n}{\underset{j=1}{\sum }}T_{i,j}\left(C\right) \end{array}$$

(7) $$\begin{array}{l} GMP=max\left(T_{0,0}\left(C\right),T_{0,1}\left(C\right),...,T_{0,n}\left(C\right),...,T_{m,n}\left(C\right)\right) \end{array}$$

Therefore, the weighted values in the height attention module, width attention module and channel attention module are calculated as in formula 8, formula 9, and formula 10, respectively.


(8) $$\begin{array}{l} H^{1*W*C}=AvgPool\_H(f^{1*1}(d^{1*1}(Concat(AvgPool\_H(T^{H*W*C}),\\ MaxPool\_H(T^{H*W*C}))))) \end{array}$$

(9) $$\begin{array}{l} W^{H*1*C}=AvgPool\_W(f^{1*1}(d^{1*1}(Concat(AvgPool\_W(T^{H*W*C}),\\ MaxPool\_W(T^{H*W*C}))))) \end{array}$$

(10) $$\begin{array}{l} C^{1*1*C}=W^{1}\left(W^{0}\left(GAP\left(R^{H*W*C}\right)\right)\right)+W^{1}\left(W^{0}\left(GMP\left(R^{H*W*C}\right)\right)\right) \end{array}$$

where f represents the 1*1 convolution operation, batch normalization (BN) and the fusion operation of the rectified linear unit (ReLU) non-activation function; d represents the 1*1 convolution operation, BN and the fusion operation of the ReLU non-activation function; *W*_0_ is the fusion operation of the fully connected layer and the ReLU non-activation function; *W*_1_ is the fusion operation of the fully connected layer and the sigmoid non-activation function.

For the input feature map *R*^H*W*C^, the weighted output feature map is calculated as in formula 11.


(11) $$\begin{array}{l} O^{H*W*C}=R^{H*W*C}*H^{1*W*C}*W^{H*1*C}*C^{1*1*C} \end{array}$$

#### 3.3.2. GABNet

**Figure 3:**
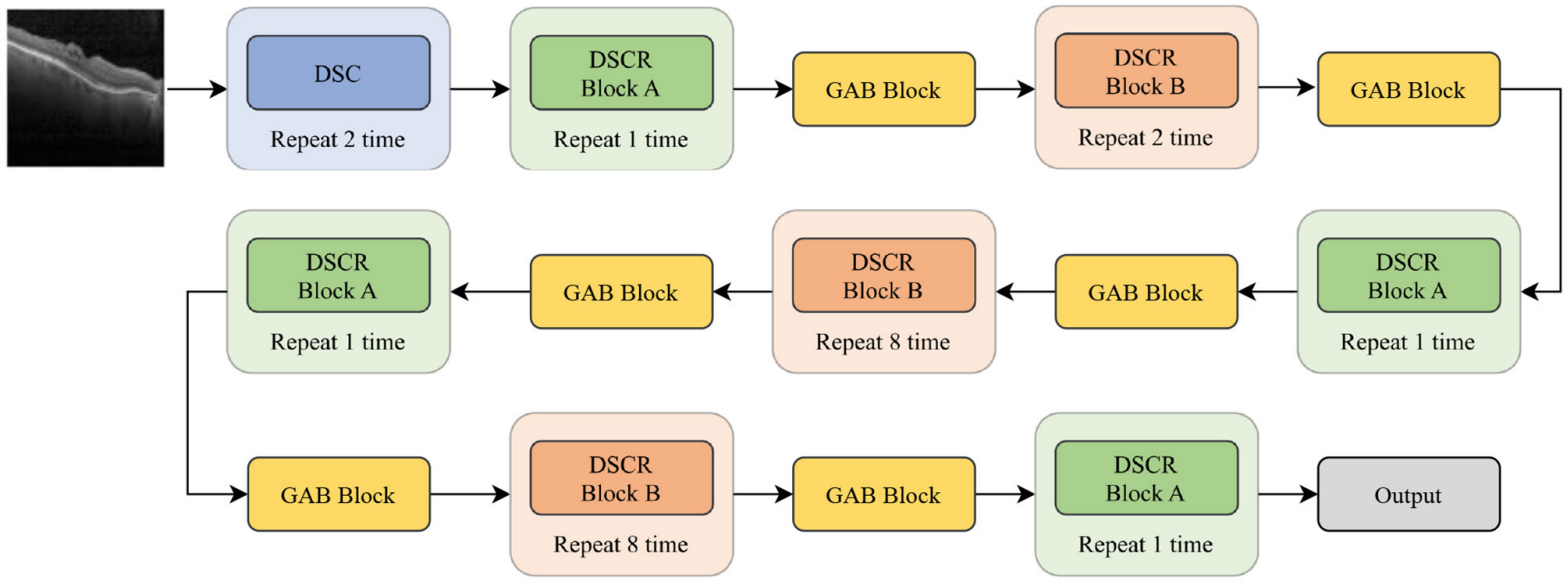
GABNet architecture.

**Figure 4:**
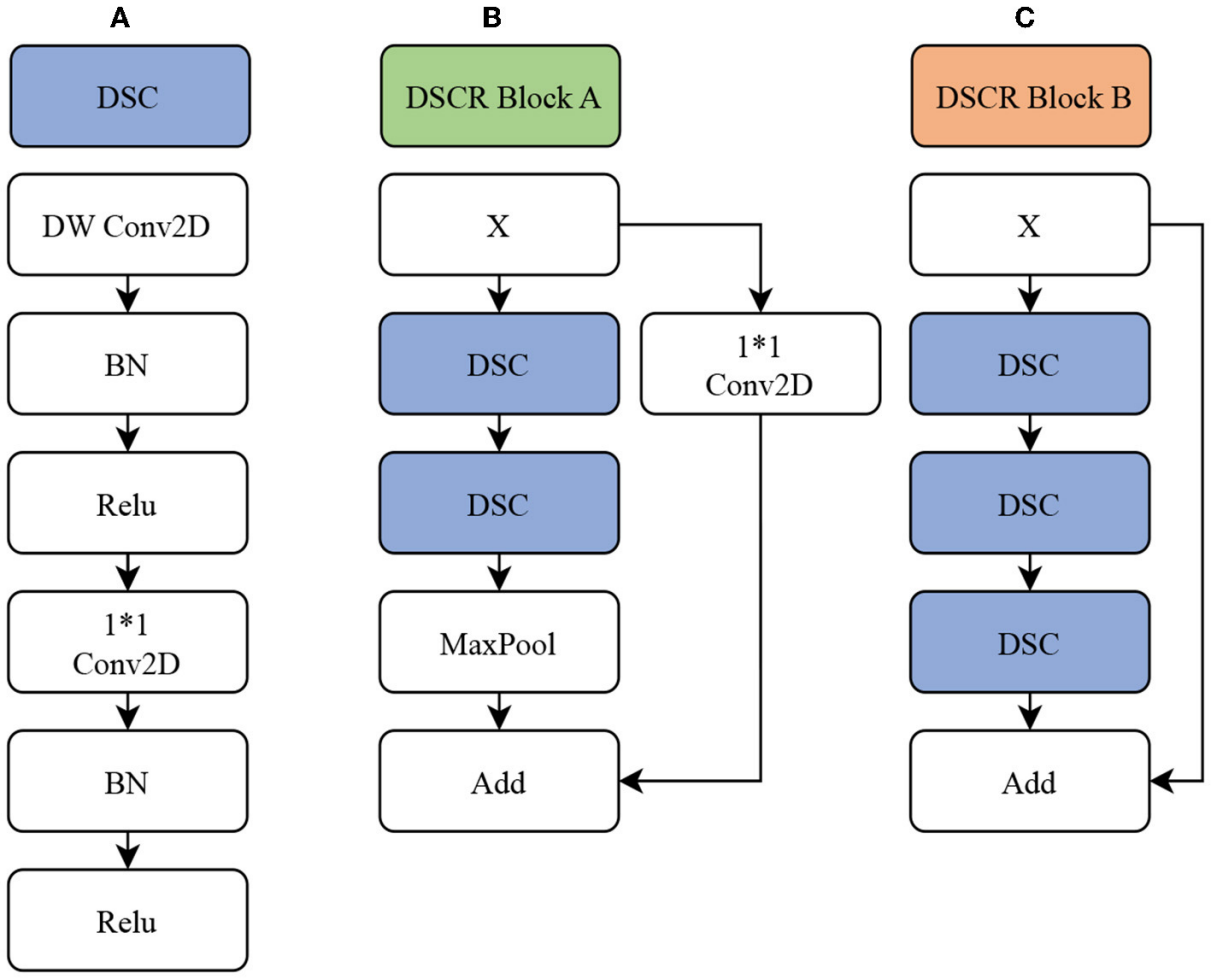
GABNet subnetwork architecture. **(A)** DSC module. **(B)** DSC residual (DSCR) Block A. **(C)** DSCR Block B. DW Conv2D, deep separable convolution layer; Conv2D, convolution layer; BN, batch normalization; ReLU, activation function; MaxPool, maximum pooling layer; Add, element-by-element addition.

In this paper, GABNet is used as a feature extraction network after data preprocessing. The overall structure of the network model is shown in Figure 3, and it can be divided into four parts: a DSC module (Figure 4A), a DSC residual (DSCR) Block A (Figure 4B), DSCR Block B (Figure 4C), and a global attention module (GAB; Figure 1 GAB). It has been shown that the DSC operation is effective in reducing the computational complexity of a network while maintaining little variation in its accuracy (Howard et al., 2019). Thus, GABNet makes extensive use of the DSC operation. The use of residual structures can effectively reduce the degradation of the network (He et al., 2016), so two modules, DSCR Block A and DSCR Block B, are constructed throughout the network. The attention mechanism is effective in improving the classification accuracy of the network when the number of parameters does not vary greatly, so the global attention mechanism is followed by an attention mechanism in each residual structure, in order to improve the effect of the network in this case.

## 4. Experiment

### 4.1. Experimental conditions

In the experimental environment of this paper, the evaluation is conducted on an Nvidia Tesla V100 with 16 GB of memory. The CUDA, cuDNN, Python, TensorFlow, and Keras versions are 11.6, 8.4.1, 3.8.8, 2.8.0, and 2.4.3, respectively.

### 4.2. Dataset

**Figure 5:**
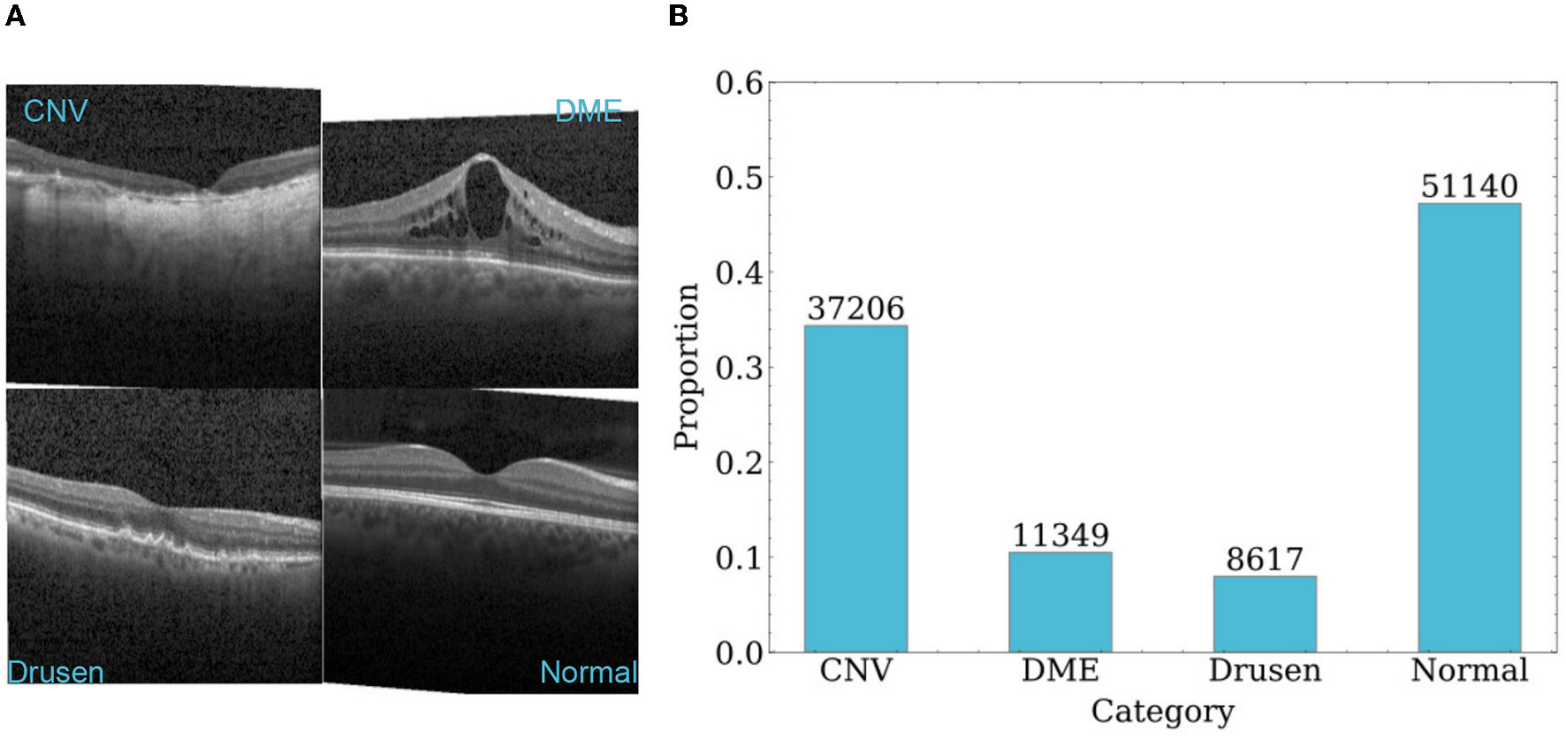
An overview of samples in dataset. **(A)** Representative OCT images of each category. **(B)** Sample size distribution of each category.

The dataset used in this paper is the UCSD retinal OCT dataset, which contains 108,312 OCT images from 4686 patients (Kermany, 2018). These images were acquired using Spectralis OCT from Heidelberg Engineering, Germany. The dataset consists of four categories: CNV, DME, drusen, and normal. The sample sizes of the four categories are 37,206, 11,349, 8,617, and 51,140, respectively. Each category has 250 samples in the test set. The dataset was collected from retrospective cohorts of adult patients by various institutions, including the Shiley Eye Institute of the University of California San Diego, the California Retinal Research Foundation, Medical Center Ophthalmology Associates, the Shanghai First People's Hospital, and the Beijing Tongren Eye Center, between July 1, 2013 and March 1, 2017. More details about the dataset can be found in the study (Kermany, 2018). Typical images and the sample size distribution of each dataset category are shown in Figure 5.

### 4.3. Evaluation criteria

To perform a quantitative analysis and obtain objective comparison results, we evaluate the diagnostic performance of the proposed approach according to the Accuracy (Acc), Recall, Precision, Specificity, F1, and area under the curve (AUC) metrics. It should be noted that the aforementioned Acc, Recall, Precision, Specificity, F1, and AUC metrics are all calculated based on weighted averages for each corresponding class, in order to obtain a comprehensive performance evaluation. Table 1 presents the confusion matrix for multi-class classification of Retinal OCT images. For each class *i*, the true positive (*TP*_*i*_), false positive (*FP*_*i*_), false negative (*FN*_*i*_), and true negative (*TN*_*i*_) can be easily obtained using the formula 12–15. The number of samples in the class i is represented by *support*_*i*. With these four values, we can calculate the Acc, F1 score, precision, specificity and recall. Acc is the proportion of correctly classified samples among all participating samples, as shown in formula 16. Recall is the proportion of correct predictions among all positive samples, as shown in formula 17. Precision is the proportion of correctly classified positive examples among all divided positive examples, as shown in formula 18. Specificity is the proportion of correctly classified negative examples among all divided negative examples, as shown in formula 19. F1 is an index used in statistics to measure the accuracy of binary classification models. It takes both model accuracy and recall into account, as shown in formula 20. The F1 score can be regarded as a harmonic average of the model accuracy and recall, whose maximum value is 1 and minimum value is 0. The AUC is the area under the receiver operating characteristic (ROC) curve enclosed by the coordinate axes, and its value range is between 0.5 and 1.


(12) $$\begin{array}{l} TP_{i}=X_{ii} \end{array}$$

(13) $$\begin{array}{l} FP_{i}=\overset{4}{\underset{j=1}{\sum }}X_{ji}-TP_{i} \end{array}$$

(14) $$\begin{array}{l} FN_{i}=\overset{4}{\underset{j=1}{\sum }}X_{ij}-TP_{i} \end{array}$$

(15) $$\begin{array}{l} TN_{i}=\overset{4}{\underset{j=1}{\sum }}\overset{4}{\underset{k=1}{\sum }}X_{jk}-TP_{i}-FP_{i}-FN_{i} \end{array}$$

(16) $$\begin{array}{l} Acc=\frac{\overset{4}{\underset{j=1}{\sum }}X_{jj}}{\overset{4}{\underset{j=1}{\sum }}\overset{4}{\underset{k=1}{\sum }}X_{jk}} \end{array}$$

(17) $$\begin{array}{l} Recall=\frac{\overset{4}{\underset{i=1}{\sum }}\frac{TP_{i}*support\text{\_}i}{TP_{i}+FN_{i}}}{\overset{4}{\underset{i=1}{\sum }}support\text{\_}i} \end{array}$$

(18) $$\begin{array}{l} Precision=\frac{\overset{4}{\underset{i=1}{\sum }}\frac{TP_{i}*support\text{\_}i}{TP_{i}+FP_{i}}}{\overset{4}{\underset{i=1}{\sum }}support\text{\_}i} \end{array}$$

(19) $$\begin{array}{l} Specificity=\frac{\overset{4}{\underset{i=1}{\sum }}\frac{TN_{i}*support\text{\_}i}{TN_{i}+FP_{i}}}{\overset{4}{\underset{i=1}{\sum }}support\text{\_}i} \end{array}$$

(20) $$\begin{array}{l} F1=\frac{2*Precision*Recall}{Precision+Recall} \end{array}$$

**Table 1:** Confusion matrix for retinal OCT classification.

**Confusion matrix**		**Predicted class**			
		**CNV**	**DME**	**Drusen**	**Normal**
Actual class	CNV	*X*_11_	*X*_12_	*X*_13_	*X*_14_
	DME	*X*_21_	*X*_22_	*X*_23_	*X*_24_
	Drusen	*X*_31_	*X*_32_	*X*_33_	*X*_34_
	Normal	*X*_41_	*X*_42_	*X*_43_	*X*_44_

### 4.4. Experimental results

To ensure objective and fair comparison among the algorithms, we used softmax as the activation function and cross-entropy as the loss function for all compared algorithms. We utilized adam optimizer with an initial learning rate of 0.0001 and a batch size of 10. The model was trained for 300 epochs. Moreover, we employed several optimization strategies, including scaling down the learning rate to 1/10 if the validation accuracy did not improve for 5 consecutive times, and early stopping if the model did not improve validation accuracy for 11 consecutive times.

Regarding the selection of data, we conducted experiments using two distinct approaches. The first approach involved training the models on the complete dataset, which will be subsequently referred to as “complete model” throughout this paper. The second approach consisted of training the models on a balanced subset of the complete dataset, containing corresponding 1,000 images per class, which will be referred to as “limited model” in the following sections.

On the general UCSD retinal OCT dataset, this paper evaluates the model performance in four perspectives: whether to use balanced data, different classification algorithms, whether to use transfer learning, and whether to use attention mechanisms. Our proposed GAB attention mechanism is tested against different attention mechanisms, such as SE, the CBAM and CoorDatt. The proposed GABNet is compared with the Xception and EfficientNetV2B3 algorithms. The results of the test comparisons are shown in Table 2. Compared with the general attention mechanisms proposed in recent years, such as SE, CBAM, and CoorDatt, the GAB attention mechanism is improved to some extent. The GABNet classification algorithm has demonstrated superior performance over Xception and EfficientNetV2B3, without the use of transfer learning, in terms of parameter efficiency and various classification metrics. This confirms the effectiveness of the GAB attention mechanism and the GABNet classification algorithm.

**Table 2:** Comparative evaluation of various algorithms on different datasets.

**Dataset**	**Classification algorithm**	**Transfer learning**	**Attention mechanism**	**Acc**	**Recall**	**Specificity**	**F1**	**AUC**
Limited model	Xception	No	No	0.94	0.94	0.98	0.9398	0.9937
		Yes	No	0.976	0.976	0.992	0.9761	0.9988
		Yes	SE	0.978	0.978	0.9927	0.9779	0.9988
		Yes	CBAM	0.978	0.978	0.9927	0.978	0.9979
		Yes	CoorDatt	0.985	0.985	0.995	0.985	0.9991
		Yes	GAB	**0.99**	**0.99**	**0.9967**	**0.99**	**0.9994**
		Yes	No	0.971	0.971	0.9903	0.971	0.9985
		Yes	SE	0.971	0.971	0.9903	0.971	0.9984
		Yes	CBAM	0.972	0.972	0.9907	0.972	**0.9986**
		Yes	CoorDatt	0.974	0.974	0.9913	0.9739	0.9975
		Yes	GAB	**0.976**	**0.976**	**0.992**	**0.976**	0.9958
		-	GAB	**0.954**	**0.954**	**0.9846**	**0.954**	**0.998**
Complete model	Xception	No	No	0.95	0.95	**0.9833**	0.9501	0.9961
		Yes	No	0.977	0.977	0.9923	0.977	**0.9992**
		Yes	SE	0.978	0.978	0.9927	0.978	0.9985
		Yes	CBAM	0.961	0.961	0.987	0.9612	0.9985
		Yes	CoorDatt	0.971	0.971	0.9903	0.9709	0.9985
		Yes	GAB	**0.98**	**0.98**	**0.9933**	**0.98**	**0.9992**
		Yes	No	0.968	0.968	0.9893	0.9681	0.9977
		Yes	SE	0.971	0.971	0.9903	0.9711	**0.9986**
		Yes	CBAM	0.973	0.973	0.991	0.973	0.9976
		Yes	CoorDatt	0.966	0.966	0.9887	0.966	0.9979
		Yes	GAB	**0.978**	**0.978**	**0.9927**	**0.9781**	0.9983
		-	GAB	**0.965**	**0.965**	**0.9883**	**0.965**	**0.9971**

Value in bold means the best of the same class.

“-” Means that the algorithm does not have this feature or property.

#### 4.4.1. Validity of the GAB and GABNet

In this paper, Xception and EfficientNetV2B3 are regarded as the basic classification modules, in which the input height and width of the Xception classification framework are 299. To achieve the best classification effect for EfficientNetV2B3 (Tan and Le, 2021), the height and width of the input image provided to this classification model are scaled to 300. To verify the effectiveness of the GAB attention mechanism, we test whether the GAB has any influence on the Xception and EfficientNetV2B3 classification algorithms based on the same dataset and the same algorithm. Under the limited model, the effects of Xception and EfficientNetV2B3 integrated with GAB module is 1.4 and 0.5% higher than those obtained by models without the GAB module, respectively. Under the complete model, Xception and EfficientNetV2B3 with the GAB module achieve 0.3 and 1% improvements, respectively, over the models without the GAB module.

**Figure 6:**
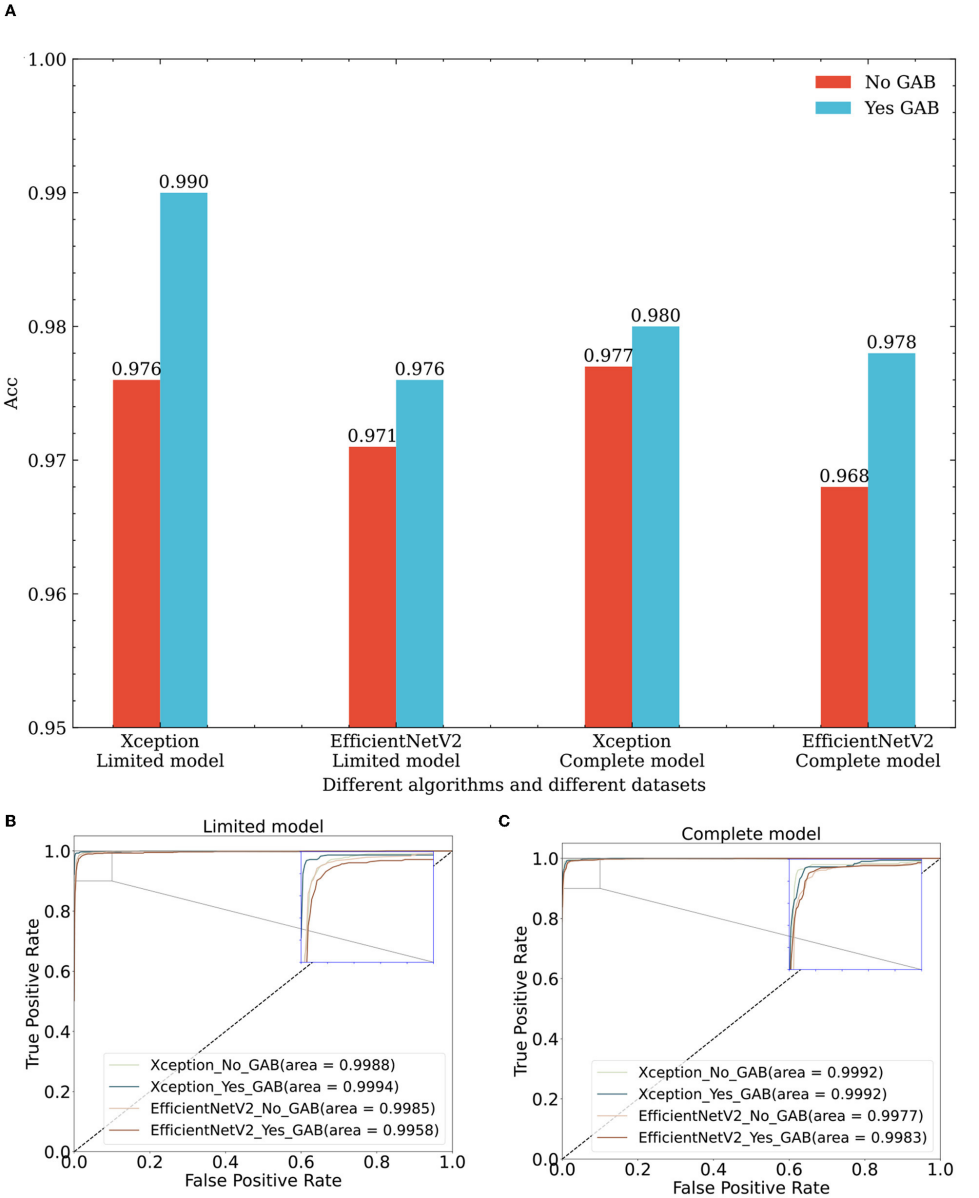
Performance comparison of classification algorithms with and without GAB on different datasets. **(A)** Effect of GAB on classification accuracy across diverse datasets and algorithms. **(B)** Effect of GAB on ROC of different algorithms in the limit model dataset. **(C)** Effect of GAB on ROC of different algorithms in the complete model dataset. Xception_No_GAB, Xception_Yes_GAB represent Xception without GAB and with GAB respectively; EfficientNetV2_No_GAB, EfficientNetV2_Yes_GAB represent EfficientNetV2 without GAB and with GAB respectively.

The GAB attention mechanism is composed of DSC operations, so it can be used as a general module and can be seamlessly connected to any network feature map. The evaluation results obtained by different algorithms on different datasets (lines 2, 6, 8, 12, 16, 20, 22, and 26 in Table 2 and Figure 6) are improved to a certain extent, thus proving the effectiveness of the GAB algorithm.

**Figure 7:**
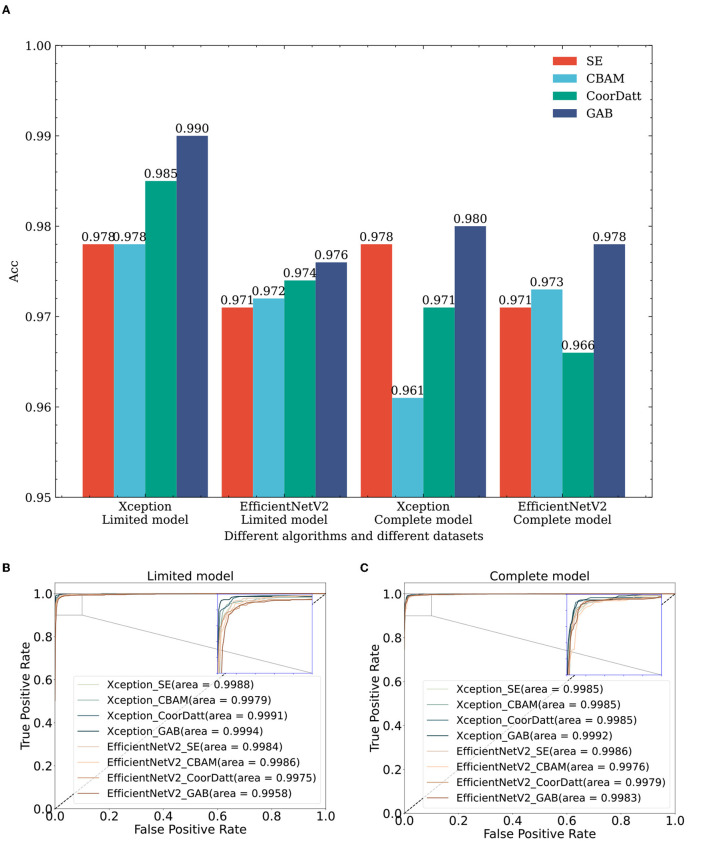
The impact of attention mechanisms on classification performance: a comparative study across multiple algorithms and datasets. **(A)** Effect of attention mechanisms on classification accuracy across diverse datasets and algorithms. **(B)** Effect of attention mechanisms on ROC of different algorithms in the limit model dataset. **(C)** Effect of attention mechanisms on ROC of different algorithms in the complete model dataset. Xception_SE, Xception_CBAM, Xception_CoorDatt and Xception_GAB represent the fusion of Xception with four different attention mechanisms, SE, CBAM, CoorDatt and GAB, respectively; EfficientNetV2_SE, EfficientNetV2_CBAM, EfficientNetV2_CoorDatt, and EfficientNetV2_GAB represent the fusion of EfficientNetV2 with four different attention mechanisms, SE, CBAM, CoorDatt and GAB, respectively.

To verify the efficiency of the GAB attention mechanism, we conduct tests on the same dataset with the same algorithm, as shown in rows 3–6, 9–12, 17–20, and 23–26 in Table 2 and Figure 7, where the sample size of each dataset category is balanced 1,000 for the limited model dataset. In comparison of different attention modules, such as SE, CBAM, CoorDatt and GAB, the Acc, F1 value and AUC results show that they all provide certain improvements over models without any attention mechanisms. Notably, the GAB attention module proposed in this paper provides improvements of up to 1.4%. Under EfficientNetV2, in the same comparison, the GAB module is 0.5% better than the original algorithm. Compared to SE, the CBAM and CoorDatt, the GAB module can effectively learn the main differences between the categories when the sample size of the dataset is small, which can maximally improve the classification effect of the model. In parallel, for the complete model dataset, the dataset is the complete dataset provided by UCSD with a large sample size but significant imbalances between categories. The Xception algorithm in the complete model with four attention mechanisms is reduced compared to that in the limited model. The unbalanced training dataset of the model has an impact, resulting in the model having inconsistent recognition abilities for various categories and being biased toward categories with large sample sizes. The reason for that may be the Xception model has some limitations regarding its prediction ability for cases with small sample sizes. EfficientNetV2B3 produces little difference between the prediction results obtained on the two different datasets, indicating that it has little influence on the dataset differences and has some resistance to interference.

**Figure 8:**
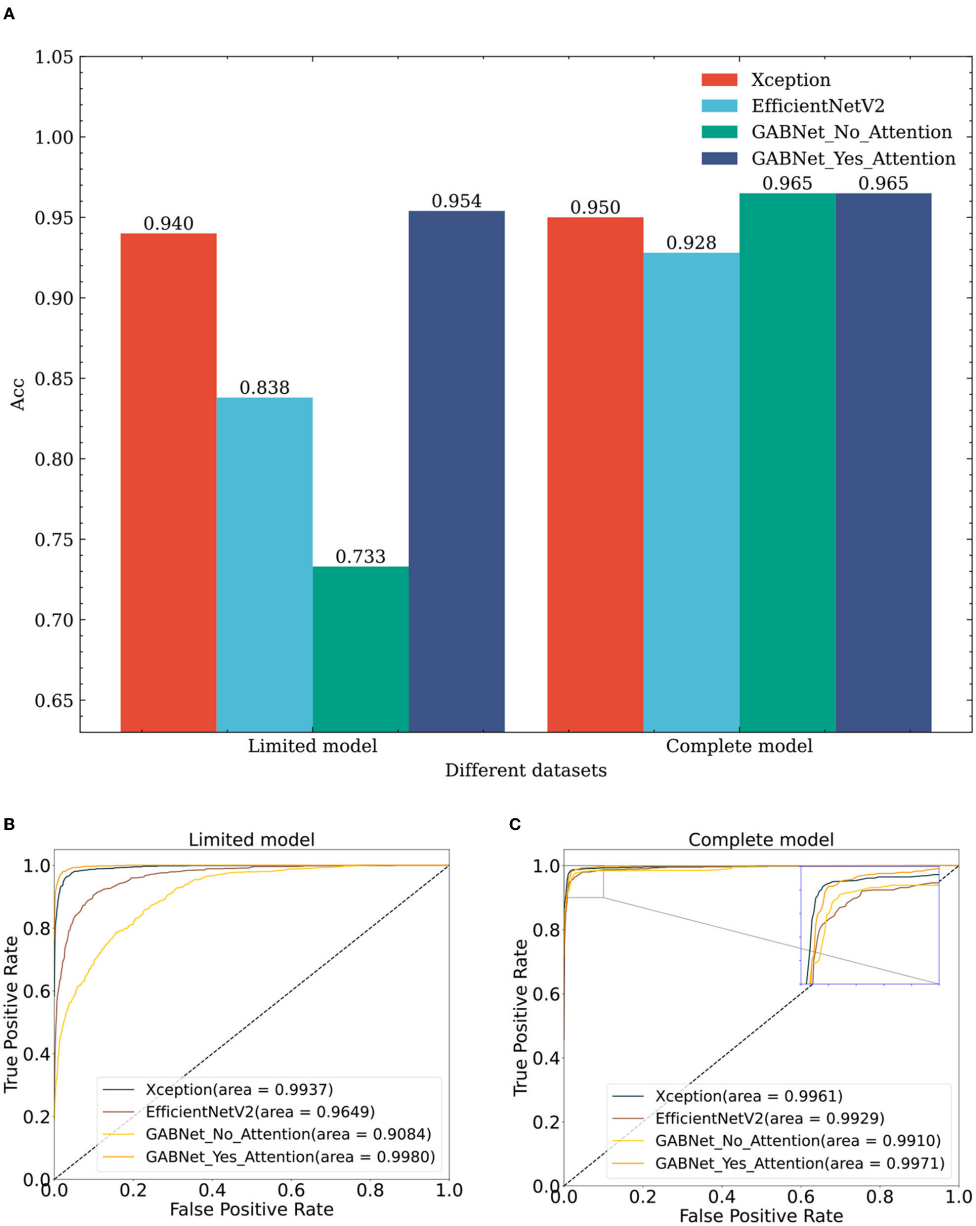
Comparing classification results of different algorithms on diverse datasets. **(A)** Effect of different algorithms on classification accuracy on different datasets. **(B)** Effect of different algorithms on ROC obtained on limit model dataset. **(C)** Effect of different algorithms on ROC obtained on complete model dataset. GABNet_No_GAB, GABNet_Yes_GAB represent GABNet without GAB and with GAB respectively.

Similarly, to verify the effectiveness of the GABNet algorithm, comparison experiments are conducted on different classification algorithms with same datasets. For this paper, the comparison models Xception and EfficientNetV2B3 were chosen. These models used their respective network architectures without utilizing “ImageNet”-based initialization of the weight parameters. Instead, they were initialized with random values for their weight parameters. The number of parameters required by the proposed GABNet in this paper is significantly lower, as can be observed from the numbers of network parameters presented in Table 3. Specifically, the GABNet requires 0.44 and 0.72 times fewer parameters compared to Xception and EfficientNetV2B3, respectively, indicating a substantial reduction in the number of parameters. From a dataset perspective (Figure 8; lines 1, 7, 13–15, 21, and 27–28 in Table 2), if all three comparison algorithms use random initialization parameters, the results obtained with the complete model are much better than those of the limited model, where random network parameter initialization requires more image data to be fitted for optimization purposes. Under the limited model, GABNet improves by 1.4% over Xception and by 11.6% over EfficientNetV2B3. The small sample size in the training dataset has a large impact on the nonmigratory learning abilities of Xception and EfficientNetV2B3, and their training processes have difficulty in reaching saturation, so their prediction effects drop sharply. In contrast, GABNet still maintains a high prediction accuracy in this situation. With a larger sample size of the complete model dataset, all four algorithm models have been improved, especially GABNet without the attention module, which has increased its accuracy by 23.2%. The GABNet model with the attention module achieves an improved prediction effect with the completed model over that obtained with the limited model, but the improvement is not very significant, indicating that GABNet is able to learn the main differences between the categories even when the sample size is small.

**Table 3:** Evaluation of different algorithms on retinal OCT classification.

**Algorithm**	**Acc**	**Recall**	**Specificity**	**F1**	**AUC**	**Parameters (M)**	**Test time**
ResNet50^a^(He et al., 2016)	0.941	0.941	0.9803	0.9411	0.9923	23.595	4.81 s±315 ms
InceptionV3^a^(Szegedy et al., 2016)	0.96	0.96	0.9867	0.9599	0.9947	21.811	**4.78 s±277 ms**
Xception^a^(Chollet, 2017)	0.95	0.95	0.9833	0.9501	0.9961	20.87	4.81 s±285 ms
EfficientNetV2B3^a^(Tan and Le, 2021)	0.928	0.928	0.976	0.9273	0.9929	12.937	5.39 s±204 ms
Huang^a^(Huang et al., 2019)	0.884	0.846	N/A	N/A	N/A	N/A	N/A
GABNet^a^	**0.965**	**0.965**	**0.9883**	**0.965**	**0.9969**	**9.361**	7.26 s±353 ms
FN-F1-OCT^b^(Ai et al., 2022)	0.985	0.985	0.995	0.985	0.99	99.717	18.1 s±831 ms
FN-Weight-OCT^b^(Ai et al., 2022)	0.984	0.984	0.995	0.984	0.99	99.717	15.6 s±419 ms
FN-Auto-OCT^b^(Ai et al., 2022)	0.987	0.987	0.996	0.987	0.991	99.774	15.8 s±451 ms
Kermany^b^(Kermany et al., 2018)	0.961	0.961	0.987	0.961	0.99	N/A	N/A
Hwang^b^(Hwang et al., 2019)	0.9693	N/A	N/A	N/A	N/A	N/A	N/A
Sinha^b^(Sinha et al., 2023)	0.944	0.944	0.9815	0.9448	N/A	N/A	N/A
EfficientNetV2B3+GAB^b^	0.978	0.978	0.9927	0.9781	0.9983	**18.281**	5.53 s± 94.5 ms
Xception+GAB^b^	**0.99**	**0.99**	**0.9967**	**0.99**	**0.9994**	30.354	**4.88 s±240 ms**

a Non-transfer learning methods.

b Transfer learning methods.

Value in bold means the best of the same class.

“N/A” Means that the metric was not displayed in the comparison article.

To conduct an ablation experiment on the GABNet network, the enhancement effect of the attention module in GABNet is verified. For the test effect in Figure 8, GABNet improves by 22.1% on the basis of the limited model. GABNet can effectively maintain the stability of the classification index when the sample size of the given dataset is not large. In view of the difficulty in obtaining medical data at present, GABNet has application potential in certain scenarios.

The UCSD retinal OCT dataset is by far the largest open source dataset in terms of data volume for retinal classification, and thus a large number of researchers have conducted research tests on this dataset. To compare the effectiveness of the GAB and GABNet techniques proposed in this paper, we collect and download the network models that have been tested on the UCSD retinal OCT dataset over the past five years, as shown in Table 3. To ensure fairness in comparison, we conducted evaluation synthetically on Complete model and Limited model datasets using NVIDIA Geforce RTX 3060Ti with 8GB memory and a batch size of 10. We selected the model with higher accuracy for further analysis. As shown in Table 3, “Parameters (M)” represents the total number of parameters in the model, and “Test Time” represents the mean ± sd time obtained by running 5 tests per round for 10 rounds on the test dataset.

We compared non-transfer learning algorithms with transfer learning algorithms. In the comparison of non-transfer learning algorithms (Table 3, rows 1-6), the GABNet algorithm had a certain disadvantage in testing time due to its wider branch compared to other algorithms. However, with the improvement of computing performance, we believe that this gap can be further reduced. Specifically, in terms of classification performance, GABNet showed extremely strong feature extraction and classification capabilities in the comparison of non-transfer learning algorithms. In the comparison of transfer learning algorithms (Table 3, rows 7-14), the accuracy of Xception and EfficientNetV2B3 algorithms with GAB attention mechanism was greatly improved. The Ai et al. (2022) method used ensemble learning with strong feature extraction, but the Xception+GAB method proposed in this paper significantly reduced the number of required parameters while improving the evaluation metrics, indicating the improved effectiveness of the GAB attention mechanism compared to the Ai et al. (2022) method. Compared to the transfer learning algorithms proposed in the literature, the accuracy of GABNet was slightly lower, which is also the focus of future work in this paper. The algorithm will be trained on the ImageNet dataset to obtain a network with strong feature extraction capabilities, and then transfer learning will be performed on this dataset.

#### 4.4.2. External validation dataset extension

This study retrospectively collected 256 retinal OCT images from Beijing Chaoyang Hospital, Capital Medical University. The dataset comprises four categories: CNV, DME, Drusen, and Normal, with sample sizes of 59, 94, 20, and 83, respectively. The Medical Ethics Review Board of Beijing Chaoyang Hospital approved the retrospective study. We directly used the trained algorithm model to predict all image data. Similarly, we employed the preprocessing method used for the internal testing set to process the external validation dataset. The evaluation metrics for each category in the external validation dataset are shown in Table 4. As the OCT images used for training the network models were generated by a “Spectralis OCT” device, while the external dataset images were acquired from a “Cirrus HD” device, there were certain discrepancies observed in the OCT image characteristics produced by the two devices. Specifically, the Spectralis OCT equipment is known to produce images with higher clarity compared to the Cirrus HD-OCT, which might have resulted in a decline in the evaluation metrics during model testing. This is undoubtedly one of the future research directions that we plan to pursue. We aim to evaluate the generalization ability of our proposed algorithms by utilizing other locally available high-resolution image databases.

**Table 4:** Evaluation of retinal OCT classification algorithms on external dataset.

**Classification algorithm**	**Acc**	**Recall**	**Specificity**	**F1**	**AUC**
GABNet	0.8047	0.8047	0.9332	0.7988	0.9654
EfficientNetV2+GAB	0.8594	0.8594	0.9531	0.8556	0.9727
Xception+GAB	**0.9141**	**0.9141**	**0.9723**	**0.915**	**0.9914**

Value in bold means the best of the same class.

#### 4.4.3. Extension to other types of datasets

**Figure 9:**
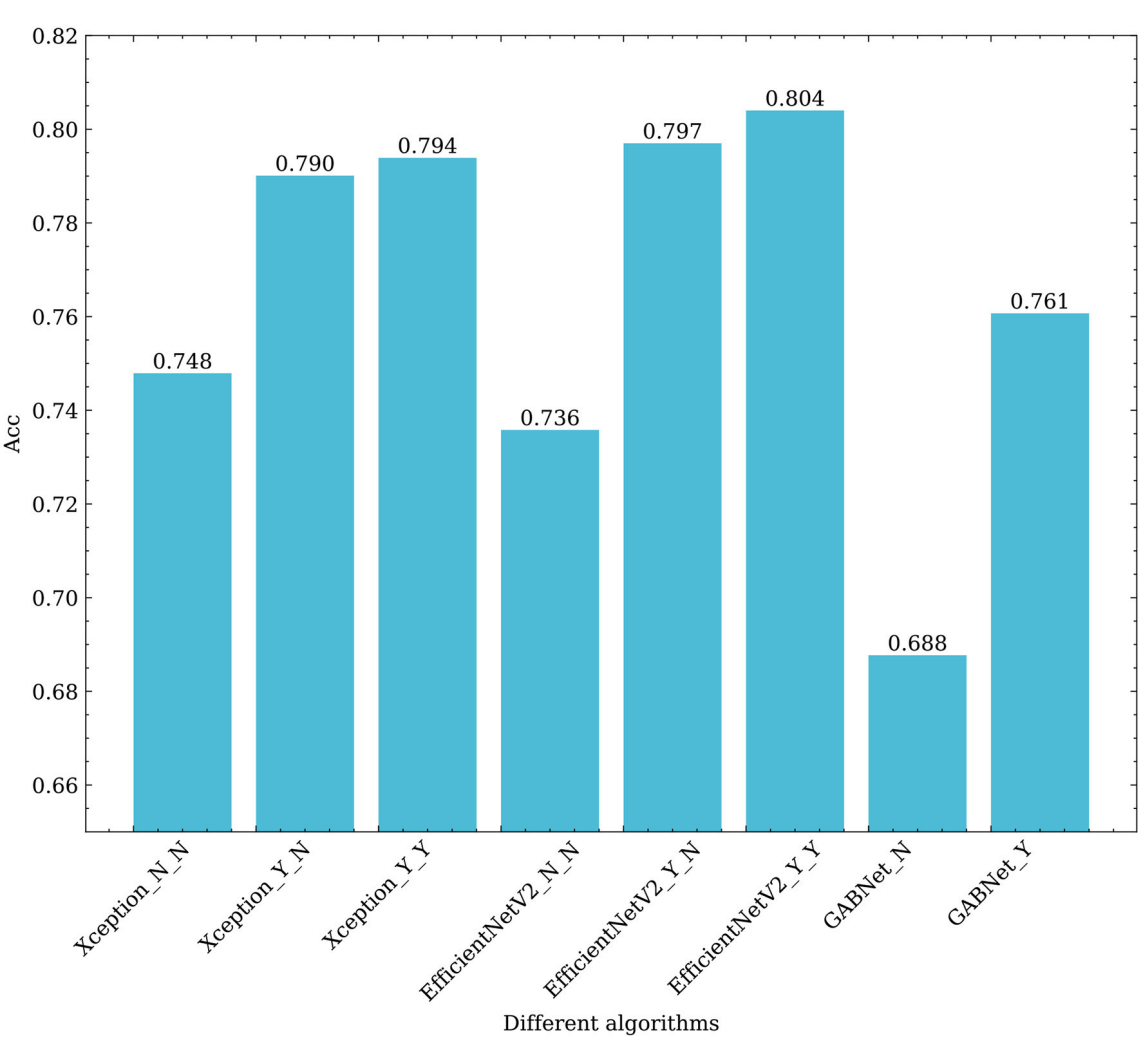
Effects of different algorithms on the classification results obtained for retinal fundus images. Xception_N_N, Xception_Y_N, Xception_Y_Y respectively represent Xception as the base classifier, no transfer learning and no GAB attention mechanism; Using transfer learning when not using GAB attention mechanism; Using transfer learning and use the GAB attention mechanism. EfficientNetV2_N_N, EfficientNetV2_Y_N, EfficientNetV2_Y_Y respectively represent EfficientNetV2 as the base classifier, no transfer learning and no GAB attention mechanism; Using transfer learning when not using GAB attention mechanism; Using transfer learning and use the GAB attention mechanism. GABNet_N,GABNet_Y represent GABNET without attention mechanism and with attention mechanism respectively.

To verify the performance of GAB and GABNet on other application scenario datasets, we selected a DR dataset for validation (Kaggle: https://www.kaggle.com/c/diabetic-retinopathy-detection/data). The dataset is taken from the Diabetic Retinopathy Detection Competition, a data modeling and data analysis competition platform. A total of 35,126 image samples are available and categorized into five categories: normal, mild, moderate, severe and proliferative DR. The sample sizes for each category are 25810, 2443, 5292, 873 and 708, respectively. Due to the severe imbalance in the dataset, this paper performs preprocessing using the data preprocessing scheme mentioned by (Ai et al., 2021). On this dataset, we use Xception, EfficientNetV2B3 and GABNet for testing, the results of which are shown in Table 5 and Figure 9.

**Table 5:** Evaluation of DR classification algorithms on fundus photograph dataset.

**Classification algorithm**	**Transfer learning**	**Attention mechanism**	**Acc**	**Recall**	**Specificity**	**F1**	**AUC**
VGG19 (Wu and Hu, 2019)	-	-	0.51	N/A	N/A	N/A	N/A
ResNet50 (Wu and Hu, 2019)	-	-	0.49	N/A	N/A	N/A	N/A
Inception V3 (Wu and Hu, 2019)	-	-	0.61	N/A	N/A	N/A	N/A
DR-IIXRN (Ai et al., 2021)	-	-	0.793	0.7933	0.8778	0.7602	0.7602
Xception	NO	NO	0.7479	0.7479	0.8307	0.6808	0.7307
	YES	NO	0.7901	0.7901	0.8767	0.7539	0.8093
	YES	YES	0.7939	0.7939	0.8711	0.7487	0.7942
EfficientNetV2B3	NO	NO	0.7358	0.7358	0.8271	0.6628	0.7127
	YES	NO	0.797	0.797	0.8776	0.7555	0.8025
	YES	YES	**0.804**	**0.804**	**0.8831**	**0.7653**	**0.8109**
GABNet	-	NO	0.6877	0.6876	0.807	0.6242	0.5987
	-	YES	0.7607	0.7607	0.8398	0.6954	0.743

Value in bold means the best of the same class.

“N/A” Means that the metric was not displayed in the comparison article.

“-” Means that the algorithm does not have this feature or property.

First, it can be seen from Figure 9 that transfer learning can greatly improve the classification ability of the associated algorithm. The accuracy of Xception and EfficientNetV2B3 are improved by 4.2 and 6.1%, respectively. This demonstrates that the efficacy of feature extraction by each network is significantly enhanced through the “ImageNet” pre-training approach, which involves utilizing large datasets and ensuring a balance across various categories. Using the GAB attention mechanism, the accuracy of Xception and EfficientNetV2B3 are improved by 0.4 and 0.7%, respectively, which shows that the attention mechanism proposed in this paper is effective and efficient. On the other hand, GABNet can also improve the classification accuracy by 3% over that of Xception and EfficientNetV2B3 without transfer learning. This shows that GABNet has certain advantages not only in retinal OCT disease classification but also in DR grading detection by fundus photograph, indicating a good generalization ability.

As shown in Table 5, it is evident that the Xception+GAB, EfficientNetV2B3+GAB, and GABNet approaches presented in this study yield enhanced accuracy compared to the methods proposed by Wu and Hu (2019) and Ai et al. (2021). While the fusion network utilized in Ai et al. (2021) demonstrated robust feature extraction, its parameter count was relatively high. In this paper, EfficientNetV2B3 and GABNet achieve improved classification correctness rates while reducing the number of required network parameters, proving that the GAB attention mechanism has a strong feature-weighted optimization function.

#### 4.4.4. Model visualization and interpretation

**Figure 10:**
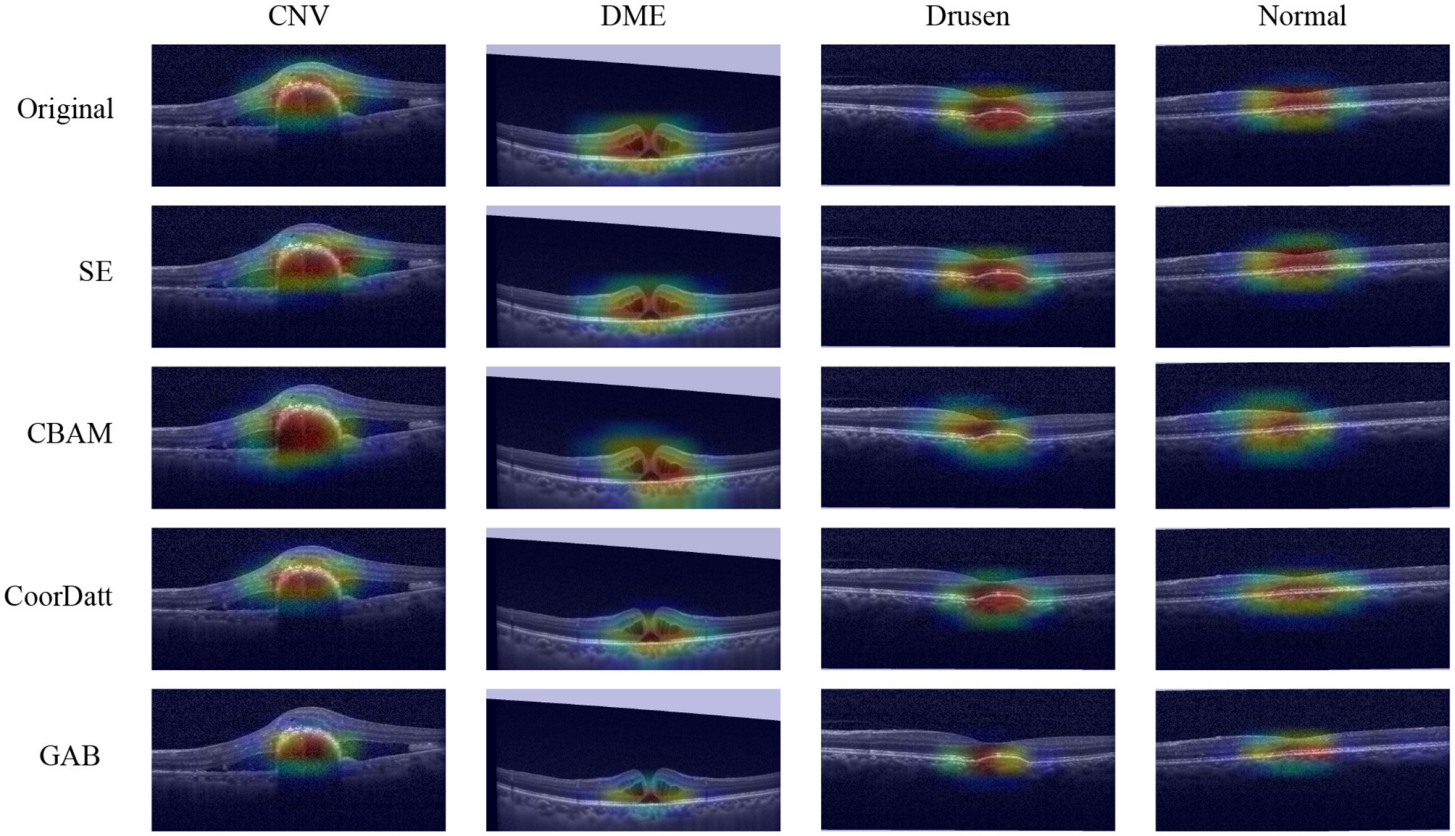
Model visualization maps of Choroidal Neovascularization (CNV), Diabetic Macular Edema (DME), Drusen and Normal by different attention mechanisms. The highlighted areas in red spectrum are considered as important in making its diagnosis.

To compare the differences of contributing regions between the proposed GAB attention mechanism and other attention mechanisms, a heatmap is created for each image using a visualization method, i.e., gradient-weighted class activation mapping (Grad-CAM). In the heatmaps (Figure 10), the most relevant category discriminating regions are highlighted in red. The fundamental purpose of heatmap generation is to construct an image that reveal the subregions of the original image to identify areas contributing to the algorithm's determination of the diagnosis. In this study, the Xception classifier is selected as the underlying model, and one image per category is arbitrarily chosen for feature visualization from the No Attention Mechanism, SE, CBAM, CoorDatt, and GAB methods.The contributing areas depicted in the heatmaps reveal that the GAB method highlights smaller regions compared to the Original, SE, CBAM, and CoorDatt methods, focusing predominantly on the lesion areas while disregarding other irrelevant regions. This substantiates the efficacy of the GAB attention mechanism. The locations highlighted by the GAB heatmap are partially consistent with human experts' experience which means good interpretation of this model.

## 5. Conclusion

This study presents a novel and effective Global Attention Block (GAB) for feedforward CNNs. The GAB is a versatile module that can be easily integrated into any CNN to improve its classification performance. Compared to commonly used attention mechanisms in current research, the GAB is shown to better focus on lesion locations in retinal OCT images, leading to improved classification results. Based on the GAB, a lightweight classification network model called GABNet is proposed, which demonstrates superior performance while also having a smaller number of parameters. Our future work includes testing the proposed algorithm on a larger set of locally sourced clinical image databases and optimizing it accordingly to improve the performance. Additionally, the use of a larger image classification database, such as ImageNet, is planned for training, and the transfer learning of the obtained GABNet classification model to more application scenarios will be expected to verify the algorithm's robustness.

## Data availability statement

The datasets generated and analysed during the current study are available from the corresponding author upon reasonable request. All deep learning methods are implemented by using TensorFlow (https://tensorflow.google.cn/). The custom script for this study will be available at https://github.com/YHHAZ/GABNet. Correspondence and requests for data materials should be addressed to Yaping Lu (luyaping@sinopharm.com).

## Author contributions

Conceptualization and methodology: ZA and XH. Validation: ZA, XH, QW, BH, YL, and FZ. Funding acquisition: FZ, XH, HW, and YL. Writing—original draft: ZA and YL. Data collection: HW, CS, JF, and YT. All authors have read and agreed to the published version of the manuscript.
